# Editorial: Advances in DYRK1A syndrome: underlying mechanisms, disease models, and novel therapeutic approaches

**DOI:** 10.3389/fnins.2026.1803346

**Published:** 2026-02-19

**Authors:** Oliver K. Glass, Amelie Piton, Anna Pfalzer

**Affiliations:** 1 Division of General Internal Medicine, Department of Medicine, Duke University, Durham, NC United States; 2 Team Genetics and Pathophysiology of Neurodevelopmental Disorders INSTITUTE OF GENETICS AND MOLECULAR AND CELLULAR BIOLOGY. (IGBMC), Illkirch-Graffenstaden, France; 3 Molecular Genetic Unit, Strasbourg University Hospital, Strasbourg, France; 4 Strasbourg Translational Research on the Autism Spectrum and Neurodevelopmental Disorders, Strasbourg, France; 5 COMBINEDBrain, Brentwood, TN, United States; 6 Department of Neurology, Vanderbilt Medical Center, Nashville, TN, United States

**Keywords:** DYRK1A, gene dosage and expression, human neural progenitors, neurodevelopmental disorders, nucleotide excision repair (NER), skeletal health

## Introduction

DYRK1A syndrome is a rare, severe neurodevelopmental disorder (NDD) characterized by intellectual disability, impaired speech development, microcephaly, craniofacial dysmorphisms, autism spectrum syndrome, and anxious/stereotypical behaviors (van Bon et al., 1993). DYRK1A syndrome is caused by disruptions to the *DYRK1A* gene located at chromosomal region 21q22.13 (van Bon et al., 1993, 2011; Møller et al., 2008) which encodes for dual-specificity tyrosine-phosphorylation-regulated kinase 1A (DYRK1A). *DYRK1A* is a highly dosage-sensitive gene (Duchon and Herault, 2016; Atas-Ozcan et al., 2021). DYRK1A syndrome is one of the most frequent monogenic causes of intellectual disability (affecting around 0.3% to 0.5% of cases) (van Bon et al., 1993; Courcet et al., 2012; O'Roak et al., 2014; Fitzgerald et al., 2015).

DYRK1A syndrome was first identified as a unique syndrome in 2008–2011 (van Bon et al., 2011; Møller et al., 2008) but knowledge of the disorder has expanded rapidly. To date, over 260 unique cases of DYRK1A syndrome have been described in the literature (Morison et al., 2022; Infantino et al., 2022; Fenster et al., 2022; Kurtz-Nelson et al., 2023; Cai et al., 2023; Ge et al., 2024; Moroni et al., 2023; Oliveira et al., 2024; Zhou et al., 2023; Huang et al., 2023; Obara et al., 2023; Lin et al., 2025; Le May et al.; Taşdelen et al., 2025; Whitaker and Serrano, 2024) and the DYRK1A Syndrome International Association maintains a registry of over 970 patients representing 61 countries (Families, 2025).

In 2024–2025, *Frontiers in Neuroscience* hosted a Research Topic: *Advances in DYRK1A Syndrome: Underlying Mechanisms, Disease Models, and Novel Therapeutic Approaches*. Its goals were to encourage publications that (1) explore the biological mechanisms of DYRK1A in cellular systems; (2) investigate domains such as skeletal health and executive functioning; and (3) advance translational opportunities. The five articles collected here advance these goals through clinical, mechanistic, and conceptual contributions.

### DYRK1A mechanism: dosage-sensitive check points

Cisternas et al. provide a comprehensive review of the role of DYRK1A dosage in the neuron-astrocyte axis, framing *DYRK1A* as having a Goldilocks zone. DYRK1A is central to neuronal morphogenesis and synaptic transmission, by acting as a checkpoint that ensures the proper number of neurons and astrocytes have differentiated at each stage of development. DYRK1A is thought to influence the astrocytes' neuroprotective activity by modulating astrocyte reactivity and glutamate excitotoxicity and viability. Cisternas et al. integrate the existing literature to hypothesize potential molecular mechanisms by which aberrant phosphorylation of DYRK1A substrates contribute to astrocyte pathology: impairing astrocyte reactivity, decreasing astrocytic uptake of glutamate to cause excitotoxicity, disrupting astrocyte regulation of appropriate Aβ levels, and contributing to tau hyperphosphorylation and aggregation. The review underscores the need for integrating the existing knowledge of DYRK1A in over-and under-expression disease contexts, so that we can advance the rational design of therapies tailored to target DYRK1A-subtrate interactions in both neurons and astrocytes.

### DYRK1A in development: human neural progenitors

Courraud et al. used IP-MS to map the DYRK1A interactome in human neural stem cells (hNSCs). They identified 35 DYRK1A interactors, 20 of which were novel. Overall, there was significant enrichment in proteins involved in cell cycle regulation and ubiquitination—notably, members of the anaphase-promoting complex and RNF114 (ZNF313). To investigate the consequences of DYRK1A loss on the transcriptome, Courraud et al. conducted siRNA knock down (KD) of *DYRK1A* in hNSCs and performed mRNA sequencing. They identified 91 significantly differentially expressed genes, with negative enrichment of genes related to the extracellular matrix and calcium binding, and upregulation of members of the early growth factor family and their downstream targets. *DYRK1A-*KD hNSCs leads to decreased proliferation and ERK activation. Importantly, Courraud et al. identified *DCAF7, GSPT1*, and *PTBP2* as novel candidate genes for involvement in NDDs. Altogether, this study underscores DYRK1A's molecular role in development and highlights common molecular pathways between DYRK1A syndrome and other NDDs.

### Clarifying diagnosis: NER look-alikes

Le May et al. describe 11 individuals initially suspected of nucleotide excision repair (NER) disorders—Cockayne syndrome (CS) and trichothiodystrophy—who were ultimately diagnosed with DYRK1A syndrome. Shared features were microcephaly, intellectual disability, feeding difficulties, ataxic gait, and deep-set eyes. However, all these patients displayed features that are not typical in NER disorders: severe language impairment, febrile seizures, and anxious or autistic behaviors. Further, all 11 patients were found to be indistinguishable from the previously published cases in DYRK1A syndrome as measured on a 20-point clinical DYRK1A scale. After UV exposure, DYRK1A patient fibroblasts did not exhibit CS-like NER defects, nor did they show the CS transcriptional signature: *ATF3* upregulation and ATF3-dependent gene downregulation. Crucially, *DYRK1A* itself is an ATF3-dependent gene downregulated in CS cells; therefore, the authors postulate that these disorders may have a common underlying cellular pathophysiology. Based on their findings, Le May et al. strongly advise that the differential diagnosis for clinicians suspecting patients of NER disorders includes DYRK1A syndrome.

### Skeletal health

Otte and Roper review skeletal phenotypes across human case reports and mouse models of DYRK1A syndrome and Down syndrome. They outline similar clinical skeletal phenotypes, including short stature, craniofacial dysmorphology, microcephaly, fetal growth restriction, and dental abnormalities. Mouse models of these disorders exhibit skeletal deficits analogous to humans, including craniofacial dysmorphisms, abnormal dentition, and a lowered body mass index. Interestingly, the authors reported potential sex differences in the Dyrk1a^+/−^ mouse, showing reduced trabecular bone thickness, density, and area in male mice but not in females. Finally, the authors list potential pathways by which DYRK1A dysregulation impacts skeletal health, including PI3K/AKT/mTOR, NGF, and REST. Overall, this review emphasizes that DYRK1A mouse models are an important research tool to characterize the skeletal anomalies in DYRK1A syndrome. This understanding will aid in future development of targeted therapies for patients with both disorders.

### Phenotype to practice: beyond cognition

Rea et al. evaluate executive functioning in 29 individuals with DYRK1A syndrome, finding consistent challenges in working memory, planning/organization, self-monitoring, and attention. While executive functioning in individuals with DYRK1A syndrome was impaired overall, many individuals exhibited differing levels of executive functioning within and across each subdomain. Importantly, the study emphasizes that most standardized executive functioning assessments are not feasible in this population, underscoring the need for developing new outcome measures that accommodate mental-age differences, minimize linguistic and motor demands, and avoid diagnostic overshadowing with conditions such as ADHD.

## Conclusion

Across the Special Topic, DYRK1A dose sensitivity emerges as the central organizing principle. Small deviations in DYRK1A activity influence progenitor proliferation, neuronal differentiation, astrocyte responses, circuit maturation, craniofacial and skeletal development, and executive functioning.

The collective work provides an initial direction for translational neuroscience: human-relevant models that may help guide clinical outcome measures, improve diagnostic pathways, and identify potential interventions.
